# Useful public health countermeasures to control the current multicountry outbreak of Monkeypox disease

**DOI:** 10.3389/fpubh.2022.1060678

**Published:** 2023-01-12

**Authors:** Khadija Leila El Siby Diatta, Oumar Faye, Amadou Alpha Sall, Ousmane Faye, Martin Faye

**Affiliations:** Virology Department, Instiut Pasteur de Dakar, Dakar, Senegal

**Keywords:** Monkeypox disease, public health countermeasures, major threat, worldwide, emerging disease

## Abstract

Monkeypox is a viral disease endemic to some countries in Central and Western Africa. However, sporadic human cases have also been reported outside of Africa. The first human case was reported in 1970 in the Democratic Republic of Congo. Very similar to the eradicated smallpox regarding its clinical representation, the Monkeypox disease is most common in children aged between 5 and 9 years with a fatality rate ranging from 1 to 11% in Africa. During the past decade, the number of countries that reported human cases of the disease grew significantly, while experts still sought knowledge on the characteristics of the virus. The recent increase in Monkeypox cases in many countries raises the concern about a possible global health threat. There is a need to subsequently provide insights into the incidence of Monkeypox disease and come up with mechanisms to prevent its emergence and contain its spread. Furthermore, it is crucial to have a better view of the global diagnostic capacity of the Monkeypox virus. This review aims to assess useful public health countermeasures to control the current multicountry outbreak of Monkeypox disease. Articles were searched in PubMed and Google Scholar electronic databases on 30 June 2022, using selected keywords, without language and date restriction. A total of 44 scientific records were published between 1 January 1962 and 30 June 2022. Herein, we discuss the epidemiological and public health situation at a global scale, provide an updated overview and data of utility for a better understanding of knowledge and research gaps in the epidemiology of the Monkeypox disease, and give useful measures for controlling the current multicountry outbreak.

## 1. Introduction

Human Monkeypox is a zoonotic disease that represents a public health concern in Central and Western Africa (1). It is caused by the Monkeypox virus (MPXV), first isolated in a colony of Asian monkeys (*Macaca facsicularis*) in 1958 at a research facility in Denmark (2). Until 1969, the disease was only detected in animals in Africa. However, in 1970, the first human case was reported in the Democratic Republic of Congo (DRC) during a period of intensified effort to eliminate smallpox, from a child who presented with skin rash and lesions (1). Belonging to the Poxviridae family and the *Orthopoxvirus* genus, the MPXV has become endemic in the DRC since its first isolation (3).

The MPXV genome is a linear double-stranded DNA of approximately 197 kb, consisting of ≈190 non-overlapping ORFs with a length >180 nucleotides (nt). The viral genome encodes for a polyprotein that is flanked by variable untranslated ends that contain inverted terminal repeats (ITRs) (4). Though it is characterized by smallpox-like symptoms, the MPXV is neither a direct ancestor nor a direct descendant of the variola virus (5).

The virus transmission to humans occurs through direct contact with body fluids and skin lesions from an infected animal (animal-to-human) or human (human-to-human) or indirect contact with contaminated lesion materials or specimens (6). The infection in humans generally manifests with mild symptoms including fever, headache, muscle aches, chills, backache, rash, and exhaustion. However, unlike the smallpox virus, the MPXV also causes lymph nodes to swell (lymphadenopathy) (7).

The MPXV presents genetic diversity with two phylogenetic clades (3). The central Africa clade is the most severe with a higher human-to-human transmission rate and is endemic in the DRC (6). The Western African clade only accounts for 3.6% of the fatality rate (8).

The MPXV was first identified outside of Africa in 2003, when a shipment of rodents from Ghana was introduced in the United States (US), causing its first outbreak in the Americas (9). More recently in 2019, the first Asian case was identified in Indonesia and imported from Nigeria (10). Between 25 and 31 May 2021, two confirmed human cases were also registered in the United Kingdom (UK) from Nigeria (11). Beyond these incidents, the MPXV was also reported in Cameroon, Cote d'Ivoire, Gabon, Liberia, Israel, Sierra Leone, and Sudan. Thus, the increasing number of human MPXV cases during the past 5 years proves that its transmission outside of Africa has become more important. This trend demonstrates that human Monkeypox disease has the potential to quickly become a major threat to global health security, hence the importance of extensive research on this virus.

In this review, we reported on the challenges and gaps in the epidemiology of human Monkeypox disease and provided some useful examples of response strategies for countermeasures and control of the current multicountry outbreak of Monkeypox disease.

Therefore, we performed a review with the aim to provide an updated overview of the current knowledge regarding Monkeypox epidemiology, its major features in terms of geographical distribution, resources for diagnosis, and medical countermeasures; exhibit examples of control strategies that allowed disease control in two different geographical contexts; and give key recommendations that could be useful for rapid control of the current multicountry outbreak.

## 2. Materials and methods

### 2.1. Search strategy

According to the PRISMA and QUORUM criteria (12), relevant articles were obtained by searching on the PubMed and the Google Scholar electronic databases as of 30 June 2022 (*n* = 957), using the search term “Monkeypox epidemiology” with no restrictions on the earliest date of the articles returned.

### 2.2. Selection criteria

Full-text original articles and detailed epidemiological reports published between 1 January 1962 and 30 June 2022 were searched. After the removal of duplicates, pertinent records were full-text screened and, if relevant, included in the Systematic Review. Documents were included if containing the following information: (i) general overview of Monkeypox epidemiology, (ii) Monkeypox disease features and distribution including phylogenetic data, (iii) Monkyepox surveillance programs including diagnostics, and (iv) Monkeypox countermeasures including available vaccines and treatment.

## 3. Results

### 3.1. Record's selection process

**Figure 1:**
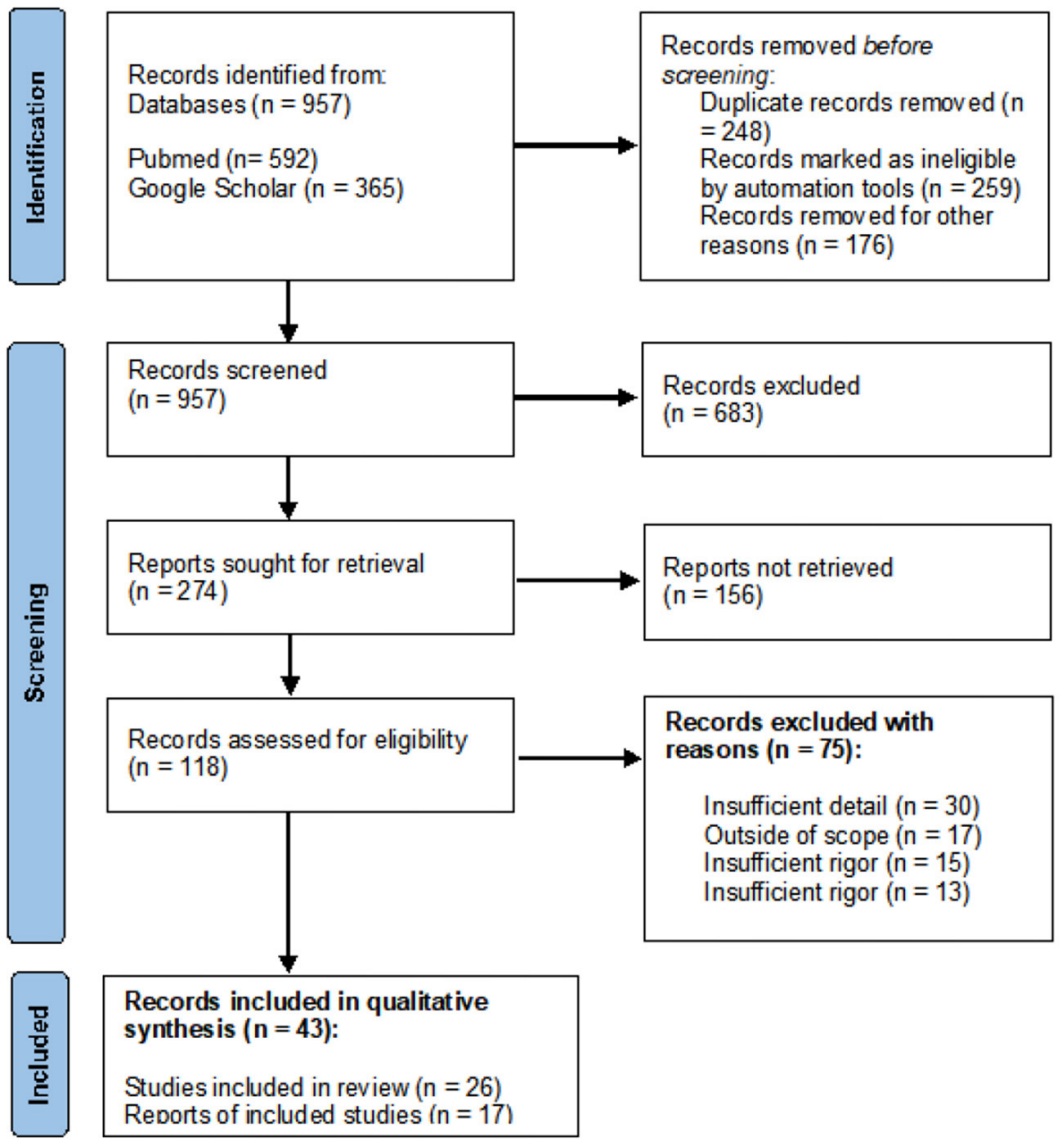
PRISMA flowchart for study selection.

Following the same eligibility criteria, a total of 274 records were identified including 259 articles and 15 reports. After duplicates were removed, the remaining 118 records were screened by title, abstract, and full text, resulting in 43 that were eligible for the final review including 26 full-text reviewed articles and 17 report citations. Relevant information reported in the identified records was summarized (Figure 1).

### 3.2. Cross-cutting issues

#### 3.2.1. Disease characteristics and epidemiology

The clinical presentation of the human MPXV identified in Central and Western Africa is similar to that of smallpox. Thus, the early signs of Monkeypox disease are often a mix of fever, myalgia, headache, sore throat, aching muscles, tiredness, chills, and backache after a mean 7 days (range 1–17) of the incubation period (7). The prodromal symptoms usually last 6–13 days, as these onset symptoms are followed by a monomorphic rash that progresses to vesicular pustular stages. The rash usually starts in the trunk, then spreads to the hands and feet, and finally, on certain occasions, to the genitalia and oral mucosa (7). It is generally followed by umbilication, the formation of scabs on the skin, and the shedding of the outer layers of the skin (7). Lymphadenopathy is another symptom of Monkeypox disease, which affects ~90% of confirmed cases (13). Among the symptoms, the swelling of the lymph nodes can either be unilateral or bilateral, or mainly touch the axillary, submandibular, or inguinal lymph nodes. In rare cases, there is a possibility of developing more severe manifestations such as soft tissue infection, coagulation disorders, pneumonitis, ocular lesions, encephalitis, and multiorgan failure (14). All those idiosyncrasies make the MPXV disease fatal in 1–11% of cases (1). Such plausible severity of the disease shows how crucial it is to have a strong epidemiological database of human Monkeypox disease. Following the MPXV prevalence in the DRC, the World Health Organization (WHO) initiated a country-wide surveillance program between 1981 and 1986, out of concern that the MPXV would become as problematic as smallpox (7). A total of 404 confirmed human cases of Monkeypox were reported in the DRC during this 6-year period (7). There were 6 human cases documented before the program began, and there was a notable decline in detected cases after the experiment ended, with the number of cases dropping from 404 to 13 between 1986 and 1992 (7).

Though endemic in some African countries, the MPXV does not spare other continents. Until 2003, the MPXV was restricted to African countries; however, with globalization, the virus was for the first time imported outside of Africa through a shipment of squirrels, Gambian rats, and African dormice that arrived in Texas from Ghana (9). The Gambian rats in the shipment were previously infected with the virus, which in turn infected prairie dogs, which then infected humans. The outbreak reported a total of 71 cases, including 35 human cases confirmed throughout 6 states (9). The symptoms were mild with a simple rash; 18 out of the 78 confirmed cases were hospitalized, and 1 of them had a severe reaction with acute headaches and painful and tonsillar lymphadenopathy (9).

In 2016, the Central African Republic (CAR) also experienced an outbreak with 26 suspected cases, 3 laboratory-confirmed cases, and 1 death (15). Several sporadic cases have also been noted throughout the years in 12 African countries where the virus is endemic (16).

The largest West African MPX outbreak in history began in Nigeria in September 2017 (17). Nigeria reported a total of 370 suspected cases in 30 states, with 45.8% laboratory-confirmed cases in 17 states (18). Men and cases aged between 31 and 40 years were the most affected (17). Overall, a total of 9 fatalities were recorded including 67% of immunocompromised patients (18). On the contrary, the 2018 outbreak in Cameroon had fewer cases, with 15 suspected cases, one laboratory-confirmed case, and no death. These cases were reported from 5 districts, and the median age of the patients was 13 years. No difference between genders has been found in Cameroon (19). Four individuals exported cases were reported from Nigeria to the United Kingdom (*n* = 2) in 2018, Israel (*n* = 1) in 2018, and Singapore (*n* = 1) in 2019, becoming the first cases of human Monkeypox exported from Africa (20). In 2019 and 2021, respectively, two other imported cases of Monkeypox from Nigeria were detected in the United Kingdom (20). In 2021, there was another imported case from Lagos State, Nigeria to Texas, United States (21). The patient developed a fever, a mild cough, and a painful genital rash on 30 June 30 (21).

There had been 7 confirmed cases of Monkeypox in the United Kingdom between 2018 and 2021 (22). As of 21 September 2022, a total of 3,552 confirmed cases of Monkeypox have been reported in the United Kingdom (22).

To date, the epidemiology of human Monkeypox is changing, and a total of 64,290 confirmed cases of Monkeypox have been reported in 115 countries outside of Africa, as of 21 September 2022 (23). However, before Monkeypox struck the West this year, some endemic countries in Africa recorded small case numbers. But contact tracing was limited as infections tend to arise in remote, forested areas, where people encounter wildlife that carry Monkeypox, such as primates and rodents.

In Africa, from January to 16 September 2022, 5,227 cases (568 confirmed and 4,659 suspected) and 137 deaths (CFR: 2.6%) of Monkeypox from eight endemic Africa Union (AU) Member States (MS): Benin (3 suspected; 3 confirmed; 0 deaths), Cameroon (29; 7; 2), Central African Republic (CAR) (17; 8; 2), Congo (14; 5; 3), Democratic Republic of Congo (DRC) (3,326; 165; 120), Ghana (535; 84; 4), Liberia (31; 2; 0), and Nigeria (704; 277; 6) and four non-endemic MS: Egypt (0; 1; 0), Morocco (0; 3; 0), South Africa (0; 5; 0), and Sudan (0; 8; 0) (24, 25).

From September 2017 to 21 September 2022, a total of 578 suspected cases have been reported in Nigeria from 32 states including 478 confirmed cases and 12 fatalities (22, 26). The year 2022 accounts for 277 of those MPXV-confirmed cases and 4 deaths from 1 January to 21 September (20, 22).

The DRC has been battling the world's largest outbreak by far, and the strain found there is also much more deadly. There were 6,216 suspected cases in September 2020, with 222 deaths (27). Children aged between 5 and 9 years were the most affected by the disease, and the most affected province was Sankuru, which is known for its rivers and rich fauna (28). Currently, MPXV is still a very common problem in the DRC, with 3,091 suspected cases and 83 deaths as of September 2021 (29) and 1,284 confirmed cases and 58 deaths between January and April 2022 (CRF 4.5%) (20).

**Figure 2:**
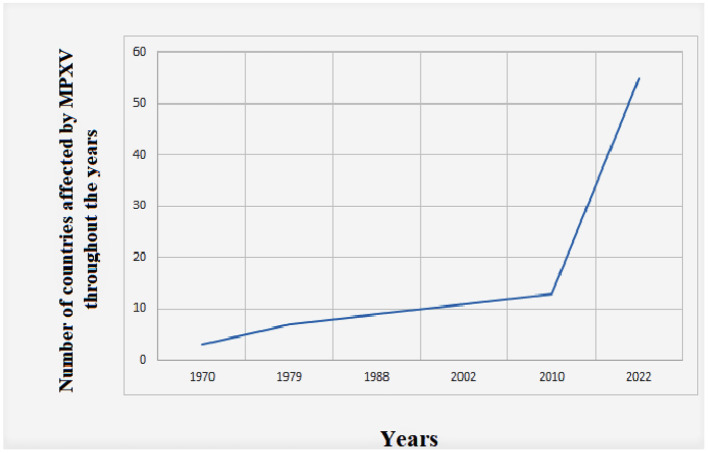
Global evolution of human Monkeypox cases throughout time from 1970 to 2022. The data were retrieved from the US Centers of Disease Control and Prevention (https://www.cdc.gov/poxvirus/monkeypox/about.html).

There has been no doubt a significant increase in MPXV cases throughout the years, and the number of affected countries is also continuously increasing (Figure 2).

**Figure 3:**
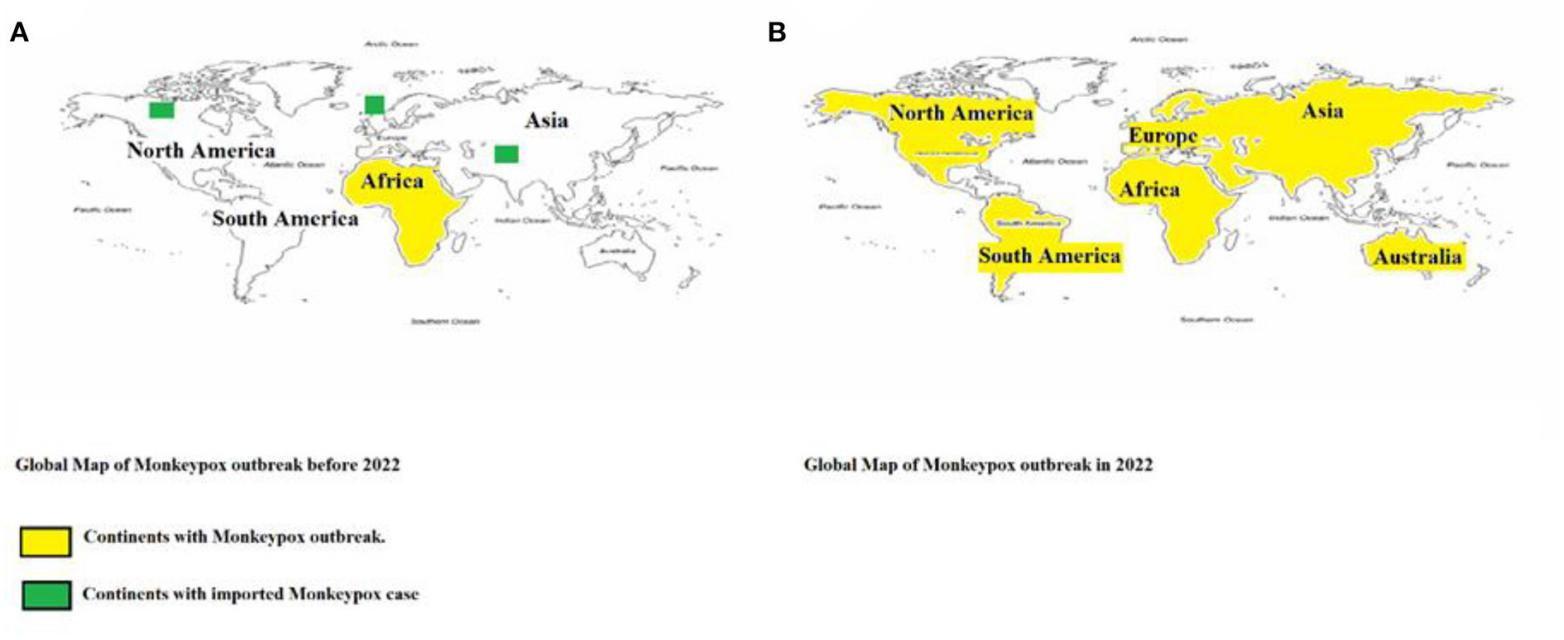
Geographical distribution of confirmed cases of Monkeypox reported **(A)** before 2022 and **(B)** during 2022. Continents that reported cases are color-coded in yellow, while the continents with imported cases are highlighted in green.

After decades of being restricted to Africa, MPXV has now been reported in every continent, except Antarctica. In 2022, the month of May was highlighted by various MPXV outbreaks around the world. In the span of 2 months, officials have confirmed over 3,000 Monkeypox cases in 42 countries (23). Within those 42 countries, only the United Kingdom, Israel, and the United States had previously reported imported (23) Monkeypox cases. The transmission goes beyond borders; it has the potential to infect anyone and anywhere, despite the difference in environments. There is a real need to inform health officers and policymakers in every country regarding this disease. Figure 3 depicts the presence of the virus on all 5 continents over the time.

In the midst of this globalization, one cannot ignore the fact that African countries, where resources are the most limited, are the most at risk. Indeed, a good number of the Monkeypox outbreaks that have been reported throughout the years usually occur in rural areas affected by armed conflicts and small villages in Africa (3). Generally, those localities are next to a tropical rainforest.

This tendency is confirmed by the experiment conducted by Levine et al., who predicted the geographic distribution of MPXV across West and Central Africa and the ecological requirements. The experiment predicted that the areas endemic for Monkeypox would generally coincide with the distribution of humid lowland evergreen tropical forests across Africa (3). These localities are usually remote, hard to access, and very limited in human and material resources. This reality is startling considering that they are the most at risk for an MPXV outbreak. Clinicians, nurses, and doctors would be overwhelmed in the face of an outbreak in those areas due to the fact that the average number of doctors per 1,000 inhabitants is usually very low in those areas.

In addition, these localities next to evergreen tropical forests are usually infested with rodents, which are the potential host reservoir for MPXV. In fact, the current data suggest that the still unknown host reservoir of Monkeypox could be a rodent prevalent in Central and Western Africa (7). Monkeypox has only been isolated from African animal species; it was first isolated in rope squirrels in 1985 and during the US outbreak in 2003 in a Gambian rat (7). Between 1970 and 1980, through an ecological investigation conducted by the WHO and the Centers for Disease Control and Prevention (CDC), several species were identified as potential host reservoirs since they were seropositive to the MPXV (7). Species such as the Congo rope squirrel, African dormice, rusty-bullied rat, and Gambian giant pouched rat could be potential host reservoirs (7). The findings above are not conclusive, meaning that the host reservoir is still unknown to the general public, which greatly limits the research and prevention that could be carried out by veterinarians, clinicians, and researchers. If the reservoir was known, it would be much easier to curate a list of animals to avoid or be wary of for the general public and veterinarians.

Despite these challenges, DRC, CAR, and Nigeria were the only countries in Africa that included Monkeypox as a notifiable disease, thus conducting a Monkeypox surveillance program by the WHO, though MPXV affects over 12 endemic countries in Africa (20). Most of the information on MPXV that could be used by scientists and researchers is being gathered in these three countries. This is a major challenge because it gives a very incomplete and biased epidemiological table for the MPXV. Moreover, the unclear picture of Monkeypox disease worldwide limited the potential actions to be taken by public health officers and policymakers. The previous data were very specific to each country, and there is no platform that synthesizes the data.

#### 3.2.2. Resources and tools for medical countermeasures (MCMs)

##### 3.2.2.1. Diagnostics

Clinically, Monkeypox disease is very similar to variola and chickenpox; thus, laboratory diagnosis is the only method to confirm cases. One of the most common procedures for the detection of MPXV in the laboratory is RT-PCR. It targets specific genes of orthopoxviruses such as E9L-NVAR (30). Another effective procedure is to use a ligand-binding assay. This assay would target B6R (the envelope protein) by using an Epoch Biosciences probe. This procedure specifically detects MPXV (30). These two methods show 100% specificity for non-variola Eurasian Orthopoxvirus and Monkeypox virus (30). Using two viral gene targets, these assays together provide a reliable and sensitive method for quickly confirming Monkeypox infections. Other genes that can be targeted in the PCR are ATI genes. The Copenhagen strain could also be used for the diagnosis using ELISA (31). Other techniques are immunohistochemistry, electron microscopy, or Western blotting tests.

**Figure 4:**
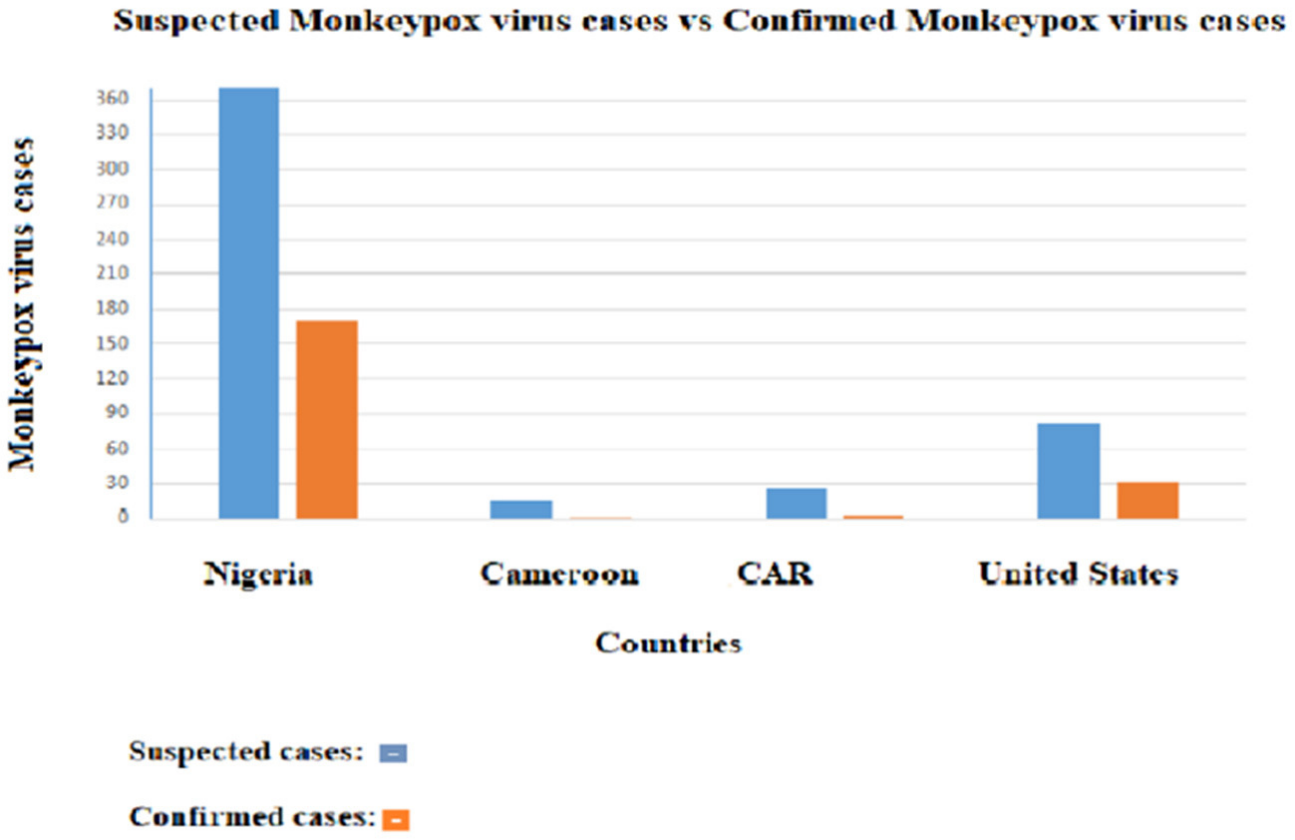
Distribution of suspected Monkeypox cases and confirmed cases in four countries. The orange bars represent the confirmed cases and the blue bars the suspected cases.

These procedures, though effective, are costly, time-consuming, and limited to professionals. In addition, the most affected areas by the disease and in need of an effective diagnosis are often secluded, with low levels of resources. A comparative analysis of suspected and confirmed Monkeypox cases between four countries shows a drastic difference between suspected and confirmed cases (Figure 4). The suspected cases are significantly higher than the confirmed cases; this difference greatly affects the response to be led by health officials. In a hospital setting, it reduces the resources to be allocated to real cases, which takes away from the effectiveness of the treatment. In addition, clinicians treating the patient have difficulties in rapidly differentiating Monkeypox disease from other rash-like illnesses, which emphasizes the importance of an efficient diagnosis method.

Poor diagnosis methods contribute to the delay in the identification of outbreaks. The earlier a suspected case is confirmed, the earlier it can be taken care of and the transmission chain limited. The rapid and accurate diagnosis of the MPXV is essential.

##### 3.2.2.2. Therapeutics

To date, there is no specific treatment for Monkeypox disease. The therapy for the disease is supportive for the most part; patients are usually hospitalized and isolated. The treatment is symptomatic.

According to the CDC, the smallpox vaccine and the antiviral drug could be used in the case of an MPXV outbreak. The antiviral drugs used are typically Cidofovir, Brincidofovir, and ST-246 (32).

- **Cidofovir**: There is no available data on the effectiveness of cidofovir in treating human Monkeypox diseases; however, through animal studies, it has proven activity against poxviruses. It inhibits DNA polymerase (32).
- **ST-246:** There has been researching done on animal species that proves the effectiveness of ST-246 for Monkeypox-confirmed patients. St-246 inhibits the release of intracellular viruses (32).

To date, there is no specific antiviral drug for Monkeypox diseases, which is currently a concern worldwide. Moreover, the disease is unknown to a good number of health workers. Besides the physical treatment of the disease, there is also a psychological side to it. Isolation can quickly become strenuous for patients.

##### 3.2.2.3. Vaccines

Vaccination is one of the fastest and most effective ways to eliminate the threat during an outbreak. The vaccines approved during Monkeypox outbreaks are smallpox vaccines such as JYNNEOS and the smallpox DNA vaccine.

- **JYNNEOS (Imvanex)**: This vaccine, also known as imvamune, is the first FDA-approved vaccine to prevent Monkeypox disease; it is a live, non-replicating smallpox vaccine. It has an effectiveness of 85% (33). This vaccine is a safer alternative to older smallpox vaccines. It is prescribed for adults aged 18 years and older and is supposed to be safe for individuals with skin disorders and immune deficiency (34). However, the vaccine is only administered to people who are at high risk for Monkeypox infection. Clinical trials using a single dose of the JYNNEOS vaccine are currently ongoing to evaluate its efficacy in humans (35).
- **Dryvax/ACAM2000**: Since the production of the smallpox vaccine ended after the eradication of smallpox (in the United States, that vaccine was called Dryvax), new stocks of the smallpox vaccine were produced in cell culture, and the vaccine was called ACAM2000 (32, 36). The ACAM2000 vaccine is licensed by the US Food and Drug Administration for immunization against smallpox disease for people determined to be at high risk for smallpox infection. It has also been recommended by the CDC as a single dose for people aged 1 year and older who have been determined to be at high risk for infection to prevent Monkeypox disease. The peak immunity is expected to be reached 4 weeks after the dose of ACAM2000 is administered (36).

We provided here a comparison of the characteristics of these two vaccine candidates (Table 1).

**Table 1:** Characteristics and differences between the two previously available vaccine candidates.

**Differences between ACAM2000 and JYNNEOS**	
Small DNA Vaccine (ACAM2000)	JYNNEOS
Live vaccinia virus	Attenuated vaccinia virus
Replicates in mammalian cells	Limited replication in mammalian cells
Single dose administration	Two dose administration
Can't be used in pregnant women or with people with history of atopic dermatitis	No such indication
licensed by the US FDA	Approved by WHO

Since the eradication of smallpox, scientists and public health authorities have kept an eye on Monkeypox, a closely related virus. As a precaution against bioterrorism, countries such as the United States maintained a supply chain of smallpox vaccines as well as an antiviral treatment thought to be effective against the virus (37). However, a failure to deploy the therapies on a large scale could probably slow down the response to tackle Monkeypox.

### 3.3. Control strategies

Considering all the previously stated facts, response activities need to be analyzed, and control strategies need to be established.

Response activities differ by country due to socioeconomic differences. For the sake of this study, two different response activities will be analyzed, one from the United States and the other from Nigeria in 2017.

In 2003, the United States experienced its first case of the MPXV. Health authorities took several steps to contain the virus. Once the MPXV case was confirmed, an emergency operation was activated (37). That operation had a domino effect on several other responses. A team of health officers, epidemiologists, and experts was deployed in Texas to help local health officers investigate possible cases of Monkeypox in both humans and animals in the United States (38). The team worked with state and federal agencies to trace the origin and distribution of potentially infected animals throughout the United States (38).

#### 3.3.1. The United States

Several guidelines and protocols were shared with the response teams: Infection control and exposure management for patients in the healthcare and community settings (38).

- Guidance on the use of the smallpox vaccine, cidofovir, and vaccinia immune globulin in the setting of an outbreak of Monkeypox. Any healthcare worker dealing with Monkeypox patients, anyone who had contact with a confirmed infected case (animal or human), and lab technicians who handled potentially infected Monkeypox specimens were advised and offered to take the vaccine.
-Guidelines for veterinarians, pet owners, animal control officers, and pet shop employees.

Besides these guidelines, they also issued an embargo and prohibited interstate transportation, sale, release, and importation of prairie dogs and rodents (38).

Any suspected or confirmed cases were taken care of in a hospital and, if needed, immediately isolated and properly treated.

#### 3.3.2. Nigeria

The first response from health officials was to deploy a team of experts to the concerned state to control the spread of the virus. In this case, the Nigerian Centre for Disease Control was the team deployed to help Bayelsa state (2). A Monkeypox response team was constituted, including clinical teams for case management, waste management, and laboratory investigations. The 12-bed medical ward of the Niger Delta University Teaching Hospital was designated as the treatment center for all Monkeypox-confirmed cases that required hospitalization (2). To help patients focus on their recovery and their health, the government announced that each Monkeypox-confirmed patient would be treated for free (2).

Several workshops were organized for the hospital staff to educate and inform them about Monkeypox, the standard use of personal protective equipment, and healthcare waste management (2).

Comparing the two responses, there is a visible need to better understand the primary challenges faced by low-resource countries, particularly those in Africa, in order to put in place the most appropriate countermeasures to stop Monkeypox disease circulation.

Developing countries usually get hit harder during an epidemic; therefore, as most countries affected by Monkeypox are among those, it is important to analyze their primary challenges.

Developing cities often lack well-equipped hospitals with large capacities to take care of several patients at the same time. The hospitals get overwhelmed at a fast pace and do not have the possibility of treating every patient who walks through their doors. There is also a real need for good equipment (such as MRI machines and oxygen tanks) and good laboratory facilities within the hospitals. In addition, many hospitals do not have an isolation ward, which is problematic since it is a viral infection that can easily be transmitted, and when the chain of transmission is not reduced, the number of cases can easily increase. As the number of cases rises, more human resources and medical equipment are needed.

There is also a lack of Monkeypox diagnostic facilities; quite often, the laboratories that have the capabilities of diagnosing the disease are limited in number and usually in big cities. This tendency is a real problem because areas affected by the outbreak are usually far from big cities and this slows down the testing process and limits it.

Besides the problem of inadequate health infrastructures, the communities can also be a challenge since a good portion of those communities have their own beliefs, and they usually put any sickness on the account of supernatural forces and would rather seek help from traditional healers. To that can be added the stigmatization that could force those needing medical assistance to avoid them at all costs while the virus is evolving in their body. Through that, the virus is evolving, and the transmission chain gets bigger.

In regards to that, effective prevention and preparedness measures need to be put in place. In order to prevent the transmission and spread of MPXV, the population has to avoid any contact with infected animals or individuals, effectively practice good hygiene at any time, isolate any suspected patients, and use personal protective equipment when caring for a patient. Despite all preventive measures, an outbreak may occur, in which case, several orientations, measures, and preparedness can be strategized. Healthcare workers could instead use a method called “ring-vaccination” to vaccinate the close contacts of confirmed cases of Monkeypox to cut off any transmission chains and contain the spread of the virus. On the basis of the recent data, the current outbreaks probably will not necessitate containment strategies beyond vaccination because, even in endemic African countries, Monkeypox is still a relatively rare infection.

A big part of preparedness is investing in an effective healthcare system. Increasing and sustaining assets to put into healthcare budgetary allocation is the path to generate a good preparedness response. The funds would help prepare the communities and train first responders and healthcare workers. With proper funding, the areas to be reinforced are.

### 3.4. The surveillance of the Monkeypox virus and diagnostic capacities

Surveillance activities provide proper data analysis to automatically alert health authorities in the face of a suspected case. Active surveillance programs for Monkeypox were ruled out in 3 of the 12 endemic countries in Africa, including Nigeria, DRC, and CAR (20).

The implementation of a well-equipped laboratory with well-trained laboratory technicians for the effective diagnosis of the MPXV in samples from suspected cases. An efficient diagnosis would allow adequate containment of the disease and reduce the possibility of transmission. Rapid diagnosis grants more tests in a shorter amount of time that allows patients to be taken care of faster with less chance of developing secondary infections. It is essential to allocate funding to developing countries to increase their diagnostic capacities on MPXV. Diagnostic capacities are usually mainly found in developed countries, while Monkeypox disease is endemic in Western and Central Africa. The gap between these two realities needs to be addressed and given proper solutions. It is crucial to implement more affordable diagnosis methods in certain areas; the recombinase polymerase amplification assay targeting the G2R gene could be an alternative. This method consists of amplifying the DNA enzymatically at a temperature range of 37–42°C in 15 min (39). The MPXV-RPA-assay has a specificity of 100% and a sensitivity of 95% (38). This technique is simple, affordable, and effective, and comes with a solar-powered mobile suitcase laboratory.

### 3.5. Research institutes

Due to the lack of substantial data on MPXV epidemiology, research is crucial and very beneficial to any country. Research on the virus grants scientists and experts to have a better image of the virus, subsequently a better understanding of its chain of transmission as well as its different clades. Studies on the two different clades would be very advantageous; they would give better insight into the virus and potentially implement a diagnosis method that would differentiate them, since the central African clade is generally more virulent, it would help health workers to give better treatments to patients. Scientists would be able to anticipate and prepare for any type of mutation. The research would advance the production of specific vaccines and antivirals. The recent emergence of the MPXV in separate populations around the world, in locations where it does not usually occur, alarmed Scientists. As of 19 May 2022, researchers in Portugal uploaded the first draft genome of the strain of MPXV that was related to the West Africa clade (40). However, more studies need to be done regarding how much the strain causing the current outbreaks differs from the one in West Africa and whether each of the outbreaks traces back to a single origin. Responses to those questions could help determine whether the sudden uptick in cases stems from a mutation that allows Monkeypox to transmit more readily than it did in the past. In addition, the detection of MPXV in people with no known contact with suspected or confirmed cases suggests that the virus might have been spreading silently.

The virus detection in men who have sex with men (MSM) also raises the question of sexual transmission of Monkeypox (41). However, no available data supports that theory (absence of the virus in vaginal fluids). Socio-anthropological studies focusing on MSM could also be promoted for a better understanding of factors driving the virus spread in this population.

### 3.6. Pharmaceutical manufacturing industries

Countries need to have the proper resources and ability to manufacture their own antiviral drugs, vaccines, and medicines. Having pharmaceutical institutes that can manufacture antivirals against MPXV would be game-changing.

### 3.7. Training medical personnel and upgrading medical infrastructure

Hospital personnel needs to be properly trained and informed about the Monkeypox disease and its clinical presentation. Monkeypox does not usually go unnoticed when it infects a person. However, if asymptomatic spread occurs as previously reported in chimpanzees in Côte d'Ivoire (42) and people close to infected animals in Cameroon and Ghana (43, 44), it will be especially troubling and could make the virus harder to track. Therefore, healthcare workers need to be better prepared.

### 3.8. Protection of lab workers

Throughout the years, it has been found that smallpox vaccination protects from Monkeypox disease. However, at this date and age, cross-protective immunity from smallpox vaccination is very rare in the population. Therefore, health officials should conduct vaccination campaigns for lab workers, veterinarians, doctors, and nurses. The available vaccines should be offered to close contact with cases to reduce the possibility of contamination and ensure that those working directly with patients and cases are protected.

### 3.9. Communication platform for experts and the public

It is important to be able to exchange information for protective measures. Adding to that was another communication platform between endemic countries so they could alert one another whenever there was a rising case of Monkeypox disease, even more so when it was in neighboring countries.

These measures have to be applied to every city in the country, not only big cities. Cities need to be self-sufficient in fighting an outbreak and containing it. This would drastically reduce the potential for transmission and, thus, the spread of the virus. When it comes to fighting an epidemic, countries should be equipped with the same resources, whether there are human, material, or expertise. The initial response of a country to an epidemic or outbreak can highly determine the impact the disease can have on the economy of a country, the consequences on the population, and how long the outbreak can last.

## 4. Discussion

The lack of knowledge on the epidemiology card of Monkeypox is an alarming gap for public health as epidemiology allows the identification of the distribution of the disease, the underlying source, and the cause of the virus emergence and methods for their control. The epidemiology data available are for the most part very specific to Nigeria and the DRC since they are two of the African countries with an active Monkeypox surveillance program with CAR (20). Our analysis showed that the increasingly high numbers of cases reported in some endemic countries during the last 5 years preceded the exponential number of cases registered in non-endemic countries. The virus is gaining ground in Central and Western Africa, while the proper resources and knowledge necessary to prevent its spread are limited. Thus, national surveillance programs are important and could allow for a real-time dashboard of the virus in each affected country, which then helps authorities put in place proper public health measures to contain the outbreak, as previously established in the DRC, CAR, and Nigeria (20). In case of an outbreak in a new place, it would be crucial for scientists to have proper epidemiology data relying on control strategies to be able to stop the circulation of the disease. There is an urgent need to establish an active surveillance program not only in each endemic country but also at the regional level, particularly in Africa. In addition, there is a need for a general platform that gathers epidemiological data worldwide and allows real-time visualization of the dashboard of suspected and confirmed cases and data analysis as in the Global Polio Eradication Initiative (GPEI) program (45).

Reliable data on the evolution of Monkeypox would help health officials control its evolution and contain it. Proper documentation of the ecological niche of the MPXV could help with a better understanding of the natural reservoir of this virus (46). It would also help to know how much of an impact the tropical environment has on the virus by using a one-health approach (47). Promoting more experimental studies could also be helpful to further characterize the clinical hallmarks of the Monkeypox disease. It would be beneficial to have a better understanding of the virus's genetic diversity and assess polymorphisms that could have driven the current international outbreak (48).

In addition, there is a need for vaccine industries to make available more vaccine doses, particularly in endemic countries. Until now, most of the cases with severe manifestations or deaths were reported from endemic countries such as the DRC, which recorded 64 deaths from January to 8 June 2020 (49, 50). As mass vaccination is not yet needed, as said by the WHO, targeted vaccination strategies with vaccines from the smallpox stockpile as well as other public health measures such as quarantine, isolation, and contact tracing can contain the outbreaks. It is, therefore, important to work on finding a standardized treatment for Monkeypox disease and develop training programs for healthcare workers that are at high risk, as well as young children, pregnant women, and immunosuppressed people (38). Training the health workers could also help them better understand the course of the disease and limit the risks of fatalities. As there is no evidence yet that it is a sexually transmitted virus, such as HIV, close contact during sexual or intimate activity, including prolonged skin-to-skin contact, maybe a key factor in the transmission. Thus, more advertisement campaigns could be promoted among gay and bisexual men on the disease presentation and help them to make contact with clinics ahead of their visit if they observe unusual rashes or lesions on any part of their body, in particular their genitalia. In addition, risk communication is crucial, as it plays an important role in avoiding homophobic rhetoric risks that could stigmatize the disease and undermine the response. In fact, efficient management in the community is vital, to give people the ease to come forward if they believe that they are infected.

The implementation of simple, affordable diagnosis techniques and diagnostic facilities would greatly help low-resource endemic countries in Africa given that those areas are the most concerned by the Monkeypox disease (51). The recent epidemiology of MPXV shows how the disease is not exclusive to African countries (50). With intercountry transportation, the disease would easily travel from one country to another. Then, early identification of cases and timely intervention in potential transmission chains are key for the rapid containment of further outbreaks (52). It is currently important that public health organizations such as WHO and CDC develop a survey to assess the capacities of diagnostics worldwide. Diagnostics companies could also produce more reagents that could help countries to rapidly detect cases and limit the disease's spread worldwide, particularly in the DRC, where the Monkeypox preparedness plan lost steam with the COVID-19 pandemic and no specific response is currently effective due to the scarcity of resources (53). The COVID-19 pandemic exposed the deficiencies of the public health system; the world was not ready for COVID-19 and it certainly is not ready for a second pandemic. It is therefore crucial to control the disease in its early stages. Given the array of challenges, it is necessary to find ways to stop the spreading of the virus and contain it in endemic countries before it becomes a global problem (54). Therefore, it is important that public health authorities in Western countries learn from the efficient control strategies used in an endemic country such as Nigeria (2).

## 5. Conclusion

According to the WHO, “*endemic Monkeypox has been reported from more countries in the past decade than during the previous 40 years*.” In its 20-year span, the virus has spread sporadically in Africa, Asia, Northern America, Southern America, Europe, and Oceania. The risk of MPXV becoming established in non-endemic countries is imminent. Most of the cases in non-endemic countries include primarily MSM. Although there are a few cases among women in that community at the moment, there is still a window of opportunity to prevent the onward spread of Monkeypox in this group. More testing and vaccines for vulnerable groups such as young children and pregnant women should be made available. The virus has been circulating and killing for decades in endemic countries in Africa, where the communities that live with the threat of the virus every day deserve the same concern, the same care, and the same access to tools to protect themselves. Ultimately, efficient control strategies, proper diagnosis methods, and patient care must be put in place and made available to every country. To support countries, the WHO could make laboratory testing and diagnosis available and provide guidance on clinical care, infection prevention and control, vaccination, and community protection.

## Author contributions

Conceived the study: MF. Analyzed and interpreted the data and wrote the manuscript: KD and MF. Revised and accepted the final version of the manuscript: KD, OumF, AS, OusF, and MF. All authors have read and agreed to the published version of the manuscript.
